# Telestroke: A Novel Approach for Post-Stroke Rehabilitation

**DOI:** 10.3390/brainsci13081186

**Published:** 2023-08-10

**Authors:** Noureddin Nakhostin Ansari, Fatemeh Bahramnezhad, Albert T. Anastasio, Gholamreza Hassanzadeh, Ardalan Shariat

**Affiliations:** 1 Department of Physiotherapy, School of Rehabilitation, Tehran University of Medical Sciences, Tehran P.O. Box 14155-6559, Iran; nakhostin@tums.ac.ir; 2 Research Center for War-Affected People, Tehran University of Medical Sciences, Tehran P.O. Box 14155-6559, Iran; 3 Department of Critical Care Nursing, School of Nursing and Midwifery, Tehran University of Medical Sciences, Tehran P.O. Box 14197-3317, Iran; bahramnezhad.f@gmail.com; 4 Department of Orthopaedic Surgery, Duke University, Durham, NC 27710, USA; albert.anastasio@duke.edu; 5 Department of Digital Health, School of Medicine, Tehran University of Medical Sciences, Tehran P.O. Box 14618-84513, Iran; hassanzadeh@tums.ac.ir; 6 Department of Anatomy, School of Medicine, Tehran University of Medical Sciences, Tehran P.O. Box 14176-13151, Iran; 7 Department of Neuroscience and Addiction Studies, School of Advanced Technologies in Medicine, Tehran University of Medical Sciences, Tehran P.O. Box 55469-14177, Iran

Despite the tremendous technologic advancements of recent years, the prevalence of stroke has increased significantly worldwide from 1990 to 2019 (a 70.0% increase in stroke and a 43.0% increase in stroke deaths). Moreover, the highest global burden of stroke is borne by low- and middle-income countries [1,2]. Rapid identification and treatment of these patients, especially in remote or rural areas, is imperative to reduce subsequent complications. The time-to-intervention for stroke is of particular importance in reducing the risk of long-term disability and mortality [3].

Virtual communication related to the distribution and provision of healthcare (referred to as “telehealth”) has become an essential component to providing equitable care and treatment services to individuals who may be unable to access care in person more readily. The telehealth concept encompasses virtual healthcare provision, communications and collaborations, research and development, education, disaster readiness, administration, and management [4]. The health resource services administration (HRSA) has defined telehealth as “the use of electronic information and telecommunications technologies to support long-distance clinical health care, patient and professional health-related education, public health and health administration” [5]. Across the literature, the terms “telemedicine” and “telehealth” are sometimes used interchangeably [6], despite a slight difference between the two terms. While telemedicine is a more limited term, referring only to remote clinical services, telehealth is a broad term which encompasses both virtual non-clinical services and clinical services [4]. As described by Khandpur et al., non-clinical aspects of telehealth can include the provision of remote training sessions, administrative meetings, and continuing health education [7].

The World Health Organization (WHO) has also commented on the emergence of “eHealth”, referring to “cost-effective and secure use of information and communications technologies in support of health and health-related fields, including health-care services, health surveillance, health literature, and health education, knowledge and research”. By this definition, eHealth can incorporate various forms of information and communication technology (ICT). Thus, applications and health promotion websites in addition to screening, assessment, and video-chat tools can all be considered forms of eHealth [8].

During the COVID-19 Pandemic, the use of telehealth became commonplace, as patients sought to avoid the risk of exposure to the viral vector. Telehealth was used to provide primary care [9] and psychiatric [10] services throughout the course of the Pandemic. Moreover, telehealth was used extensively for the prevention and treatment of various types of musculoskeletal discomforts [11]. Within the field of rehabilitation after neurological insult, the development of telestroke in 1996 has brought about a revolution in the treatment of patients with stroke. Telestroke allows physicians and advanced practice providers to begin examining and treating patients with stroke remotely utilizing various forms of technology (e.g., video conferencing, digital cameras, smartphones, tablets, and other technologies) well before reaching the hospital [12,13].

Further emphasis on education for suspectable populations, screening and monitoring for signs and symptoms of neurological change, and management advancements and insights can all continue to improve the care of patients with stroke. Improvements within the domain of telestroke can also revolutionize stroke care. Training efforts for high-risk individuals in the use of modern technologies can result in improved prevention and treatment of this disease [14]. Researchers and healthcare practitioners must consider all three levels of prevention: primary, secondary, and tertiary prevention to improve the efficiency of telestroke capabilities. Broadly speaking, primary prevention through the use of telestroke involves the careful provision of primary care through virtual platforms, including discussion regarding risk mitigation through the use of diet and exercise interventions [15] as well as pharmacotherapy targeting hypertension [16] and hypercholesterolemia [16]. Secondary prevention can also be achieved through the use of telecommunications for screening and identification of patients at risk for impending neurological insult. Patients can then follow up in the clinical setting for advanced imaging or invasive diagnostic tests. Finally, tertiary prevention once patients have been diagnosed with stroke can be at least in part administered through the use of telestroke, as patients require close follow up and rehabilitative consultations [17].

In addition to efforts geared towards the level of physicians and rehabilitative practitioners, telenursing can be effective in reducing the global burden of disease from stroke [18]. Today, the capabilities of different aspects of telehealth, such as telenursing and teleconsultation have expanded markedly [19]. Therefore, linking these entities with telestroke, especially in the discussion of primary prevention, can bring great utility in preventing the occurrence and complications of stroke, especially in developing and low-income countries.

To these aims, for this special series for *Brain Sciences* on post-stroke rehabilitation, we invite experts in the field of neurorehabilitation to submit their valuable papers including original articles and reviews. The main purpose of this issue is to highlight the novel efforts for patients who are post-stroke using new technologies. In addition, the series will include contributions using traditional methods as well. We look forward to fostering the ongoing discussion regarding the provision of stroke-related care via virtual means.
